# Supplementary material for: Twelve‑month rehospitalization after IBD‑related hospitalization in Germany: A retrospective cohort study using administrative hospital data
Source: PLoS One. 2026 Mar 31;21(3):e0346270. doi: 10.1371/journal.pone.0346270 (PMC13037979; doi:10.1371/journal.pone.0346270)
Supplement: Attachment — Submitted filename: Response to the editors_Rehospitalisation IBD.docx [file pone.0346270.s001.docx]

# 17.12.2025

# Head of Department

Prof. Dr. T. Lüdde

Phone: +49 (0) 211 81-16330

Fax: +49 (0) 211 81-18752

E-mail:[tom.luedde@med.uni-duesseldorf.de](mailto:tom.luedde@med.uni-duesseldorf.de)

**Corresponding author**

Dr. Christian Weigel

Phone: +49 (0) 211 81-16330

Fax: +49 (0) 211 81-04489

E-mail:[christian.weigel@med.uni-duesseldorf.de](mailto:christian.weigel@med.uni-duesseldorf.de)

## Clinic for Gastroenterology, Hepatology and Infectious Diseases University Hospital Düsseldorf

## Medical Faculty of Heinrich Heine University Düsseldorf

| University Hospital Düsseldorf • Moorenstraße 5 • 40225 Düsseldorf • Germany |  |
| --- | --- |

Department of Gastroenterology, Hepatology and Infectious Diseases

Kate-Lyn Maaño Claro
PLOS One

**Edits request on submission of Manuscript – Clinical and demographic factors associated with a 12-months rehospitalization among patients with inflammatory bowel disease (PONE-D-25-66720)**

Dear Kate-Lyn Maaño Claro,

we thank you for your careful review and for the opportunity to clarify the ethical aspects of our study. Please find our point-by-point responses below.

1. Please complete all items on the Human Participants Research Checklist that are relevant for your submission. There may be overlap between the checklist items and other queries listed below; please address any duplicated queries both in your response email and on the checklist itself. Upload the completed Human Participants Research Checklist as file type “Other” when you re-submit your manuscript. This document is for internal journal use only and will not be published if your article is accepted. The requested information will help us to assess whether your submission complies with PLOS ONE’s policies and adheres to applicable reporting standards. Note that your manuscript may be rejected if you provide incomplete or inadequate responses to the checklist questions and that changing the ‘Section/Category’ of your article does not affect this requirement.

*The Human Subjects Research Checklist has been completed and uploaded to the submission system as requested.*

2. Please provide additional details regarding participant consent. In the ethics statement in the Methods and online submission information, please ensure that you have specified (1) whether consent was informed and (2) what type you obtained (for instance, written or verbal, and if verbal, how it was documented and witnessed). If your study included minors, state whether you obtained consent from parents or guardians. If the need for consent was waived by the ethics committee, please include this information.

*No informed consent was obtained for this study (1/2). The study was based exclusively on fully anonymized secondary data from the IQVIA hospital database, including Section 21 datasets submitted in accordance with the German Hospital Compensation Act (KHEntgG). All data were anonymized prior to access, and the authors had no access to directly or indirectly identifiable information at any time. Re-identification of individual patients was not possible. Analyses were conducted exclusively on aggregated data. Under German law, the use of fully anonymized routine healthcare data for observational research does not require informed consent from patients, nor approval by a medical ethics committee. Accordingly, the requirement for informed consent was waived, and no consent (written or verbal) was obtained.*

*The dataset may include minors; however, as all data were fully anonymized and analyzed only in aggregate form, consent from parents or guardians was not required.* *This information has already been described in the Methods section (page 5, lines 98–111).*

3. If you are reporting a retrospective study of medical records or archived samples, please ensure that you have discussed whether all data were fully anonymized before you accessed them and/or whether the IRB or ethics committee waived the requirement for informed consent. If patients provided informed written consent to have data from their medical records used in research, please include this information

*As stated above, all data were fully anonymized; therefore, no approval by a medical ethics committee was required.*

4. Once you have amended this/these statement(s) in the Methods section of the manuscript, please add the same text to the “Ethics Statement” field of the submission form (via “Edit Submission”).

*The Ethics Statement field in the submission system contains all of the above-mentioned information.*

Thank you very much for considering our manuscript. We look forward to your response and the opportunity to contribute to the field of gastroenterology.

With kind regards,

**Christian Weigel & Sven H. Loosen**
(on behalf of all co-authors)
