# Supplementary material for: Twelve‑month rehospitalization after IBD‑related hospitalization in Germany: A retrospective cohort study using administrative hospital data
Source: PLoS One. 2026 Mar 31;21(3):e0346270. doi: 10.1371/journal.pone.0346270 (PMC13037979; doi:10.1371/journal.pone.0346270)
Supplement: Attachment — Submitted filename: Response_to_the_editors_and_reviewer_PONE-D-25-66720_auresp_2.docx [file pone.0346270.s003.docx]

# 15.03.2026

# Head of Department

Prof. Dr. T. Lüdde

Phone: +49 (0) 211 81-16330

Fax: +49 (0) 211 81-18752

E-mail:[tom.luedde@med.uni-duesseldorf.de](mailto:tom.luedde@med.uni-duesseldorf.de)

**Corresponding author**

Dr. Christian Weigel

Phone: +49 (0) 211 81-16330

Fax: +49 (0) 211 81-04489

E-mail:[christian.weigel@med.uni-duesseldorf.de](mailto:christian.weigel@med.uni-duesseldorf.de)

## Clinic for Gastroenterology, Hepatology and Infectious Diseases University Hospital Düsseldorf

## Medical Faculty of Heinrich Heine University Düsseldorf

| University Hospital Düsseldorf • Moorenstraße 5 • 40225 Düsseldorf • Germany |  |
| --- | --- |

Department of Gastroenterology, Hepatology and Infectious Diseases

Marwan Salih Al-Nimer, MD, PhD
Academic Editor
*PLOS One*

**Response to Reviewers – PONE-D-25-66720**

*Title: Twelve-month rehospitalization after IBD-related hospitalization in Germany:*

*a retrospective cohort study using administrative hospital data*

Dear Dr. Al-Nimer,

We sincerely thank you and the reviewer for the careful evaluation of our manuscript entitled “Twelve-month rehospitalization after IBD-related hospitalization in Germany: a retrospective cohort study using administrative hospital data” (PONE-D-25-66720).

We appreciate the constructive and insightful comments, which have helped us to improve the clarity, methodological transparency, and overall presentation of our work. Below, we provide a detailed, point-by-point response to all comments raised by the Academic Editor and Reviewer #1. All changes have been incorporated into the revised manuscript. A marked-up version with track changes and a clean version of the manuscript are submitted separately.

**Responses to Academic Editor Comments**

1. The title, objective, and conclusion of this descriptive study are inconsistent with the methodology.

Response: We thank the editor for this important observation. We revised the title, objective, and conclusion to ensure full alignment with the descriptive and administrative nature of the study. The revised title now reads: *“Twelve-month rehospitalization after IBD-related hospitalization in Germany: a retrospective cohort study using administrative hospital data.”*
The study objective and conclusion were updated to explicitly reflect that the analysis is based on administrative ICD-10–coded data and describes associations rather than clinically adjudicated outcomes.

2. Complete revision of the Methods is necessary. Write in detail the laboratory and clinical diagnosis included in this study.

Response: We appreciate the editor’s request for clarification. The Section 21 dataset contains exclusively ICD-10-GM diagnosis codes and does not include laboratory values, diagnostic thresholds, or information on how diagnoses were clinically established.

We therefore revised the Methods section and added the following clarification:
“All comorbidities were identified exclusively through ICD‑10‑GM codes documented in the electronic hospital records, including iron deficiency anaemia (D50), gastritis/duodenitis (K29), intestinal obstruction (K56), gastrointestinal hemorrhage (K92.2), peritoneal adhesions (K66.0), type 2 diabetes mellitus (E11), hypothyroidism (E03), volume depletion (E86), hypokalemia (E87.6), and hypertension (I10). The Section 21 dataset does not contain laboratory values, diagnostic thresholds, or information on how these diagnoses were clinically established.”

3. Revise the statistical analysis section because in the results there is mean, median,, SD. What about the p-values for Odds ratios

Response: This was also revised.

4. Figure 2 not informative and is poorly presented.

Response: We thank the editor for this constructive comment. After reconsideration, we agree that Figure 2 did not substantially enhance interpretability and partly duplicated information already presented in the tables. To improve clarity and avoid redundancy, we have removed Figure 2 from the revised manuscript.

5. References are typed inconsistently.

Response: We carefully reviewed and reformatted the entire reference list to ensure full consistency in accordance with PLOS ONE guidelines.

6. Type the references according to the PLoS ONE style.

Response: The reference list has now been revised and formatted according to the Vancouver style as requested.

**Journal Requirements**

Funding Statement: We have removed the funding-related statement from the manuscript, as requested. We confirm that no specific funding was received for this work. The Funding Statement in the online submission system remains unchanged and accurately reflects this information.

Data Availability Statement: Thank you for this important clarification. The dataset used in this study is owned by IQVIA (Frankfurt, Germany) and was provided under a contractual data use agreement with contributing hospitals. Due to legal and contractual restrictions, including data protection requirements and the proprietary nature of the dataset, the data cannot be shared publicly or deposited in an open repository. We have revised the Data Availability Statement accordingly and now provide a non-author institutional contact point at IQVIA for data access requests. Researchers who meet the criteria for access to confidential data may submit requests to the IQVIA Data Access/Compliance Office (IQVIA, Frankfurt, Germany, Mail: [info.germany@iqvia.com](mailto:info.germany@iqvia.com)). Access is subject to IQVIA approval and may require a data use agreement and associated fees.

Caption for each figure: We have revised the manuscript to include separate and detailed captions for each figure in a dedicated “Figure Legends” section.

Reference list: We carefully reviewed the reviewer comments and note that no specific additional publications were recommended for citation. Therefore, no changes were made to the reference list.

**Responses to Reviewer #1**

Comment: In the methods section, the manuscript describes the study design as "cross-sectional" despite including the temporal element of rehospitalizations. The study tracks patients for 12 months to detect rehospitalizations. This constitutes a retrospective cohort study design. I suggest updating the terminology throughout the manuscript.

Response: We thank the reviewer for this important clarification. The study indeed follows patients over time to assess 12‑month rehospitalizations and therefore constitutes a *retrospective cohort study*. We have updated the terminology throughout the manuscript accordingly.

Comment: Were there incomplete follow-ups, i.e., 12 months beyond December 2023? I suggest adding a line to clarify how were patients admitted in 2023 handled, as well as patients who died after discharge or lost to follow-up.

Response: We have added a paragraph to the Methods section explaining that follow‑up for patients discharged in 2023 may be shorter than 12 months due to the end of the observation window. Because the dataset only captures inpatient encounters, we cannot distinguish between patients who remained event‑free, died, or were hospitalized elsewhere. This limitation is now explicitly acknowledged.

Comment: Were the 49 hospitals similar in practices and operational environment? I suggest updating the regression model to cluster patients under their hospitals, or add a line of justification as to why clustering is not necessary.

Response: We appreciate this suggestion. Unfortunately, the Section 21 dataset is provided in aggregated form without patient‑level identifiers, which precludes hierarchical modeling. Additionally, all hospitals submit data under standardized national coding and reimbursement rules, reducing systematic inter‑hospital variability. We have added a justification to the Methods section explaining why clustering was not feasible and not expected to materially influence the results.

Comment: I suggest adding a line to confirm that VIF was assessed.

Response: We thank the reviewer for this point. VIFs were calculated for all predictors in the multivariable model, and all values were <1, indicating no relevant multicollinearity. This information has been added to the Methods section.

Comment: A valuable addition would be considering subgroup analyses, especially that the sample size is large and with a near-equal distribution between Crohn's disease and Ulcerative colitis.

Response: We appreciate the reviewer’s suggestion. While subgroup analyses (e.g., stratified by IBD subtype) are of clinical interest, the structure of the aggregated dataset limits the granularity of available variables. Important covariates such as disease activity, medication use, surgical history, and laboratory parameters are not available, which would limit the interpretability of subgroup models and increase the risk of residual confounding. To avoid over‑interpretation of incomplete data, we chose to focus on the overall cohort. We have added a sentence to the Discussion explaining this rationale.

We again thank the Academic Editor and Reviewer for their thoughtful and constructive feedback. We believe the revisions have substantially strengthened the manuscript. We look forward to your response and the opportunity to contribute to the field of gastroenterology.

With kind regards,

**Christian Weigel & Sven H. Loosen**
(on behalf of all co-authors)
